# Current Study of Alpha-Mangostin in Breast Cancer Therapy: Antioxidant Mechanisms and Redox Modulation from In Silico to In Vivo Studies

**DOI:** 10.3390/antiox15080998

**Published:** 2026-08-12

**Authors:** Muchtaridi Muchtaridi, Bintang Satrio Mahardika, Luthfi Utami Setyawati, Dhania Novitasari, Jasimah Jasimah, Febby Pratama, Nur Kusaira Khairul Ikram

**Affiliations:** 1 Department of Pharmaceutical Analysis and Medicinal Chemistry, Faculty of Pharmacy, Universitas Padjadjaran, Sumedang 45363, Indonesialuthfi15002@mail.unpad.ac.id (L.U.S.); dhania@unpad.ac.id (D.N.); jasimah22001@mail.unpad.ac.id (J.J.); febby24001@mail.unpad.ac.id (F.P.); 2 Research Collaboration Center for Theranostic Radiopharmaceuticals, National Research and Innovation Agency (BRIN), Jl. KM Bandung-Sumedang KM 21, Sumedang, 45363, Indonesia; 3 Doctoral Program of Pharmacy, Faculty of Pharmacy, Universitas Padjadjaran, Sumedang 45363, Indonesia; 4 Institute of Biological Sciences, Faculty of Science, Universiti Malaya, Kuala Lumpur 50603, Malaysia; nkusaira@um.edu.my

**Keywords:** α-mangostin, breast cancer, targeted therapy, in silico, in vitro, in vivo

## Abstract

Breast cancer remains a major disease burden and a leading cause of cancer-related morbidity and mortality among women, while current therapies are often limited by toxicity, drug resistance, and recurrence. α-Mangostin (AM), a prenylated xanthone derived from *Garcinia mangostana* L., has attracted considerable interest because of its antioxidant, redox-modulating, and anticancer properties, which may contribute to improved health outcomes and patient well-being. This study systematically reviewed the therapeutic potential of AM against breast cancer based on evidence from in silico, in vitro, and in vivo studies focusing on biomedical and pharmaceutical applications. Relevant articles were retrieved from the Scopus and PubMed databases using predefined keywords and selection criteria. Eligible studies were extracted, categorized, and analyzed using Microsoft Word and EndNote to assess the pharmacological actions, target interactions, delivery systems, and therapeutic outcomes associated with AM, while the quality of animal studies was assessed using the ARRIVE guidelines. Twenty-nine studies met the inclusion criteria. Computational studies demonstrated favorable AM interactions with ERα, STAT3, RXRα, CXCR4, AKT1, CTNNB1, and HSP90AA1. In vitro studies showed that AM inhibited cancer cell viability, induced apoptosis and autophagy, modulated reactive oxygen species, and suppressed metastatic and immune-evasion markers. Furthermore, nano-formulations, radiolabeled derivatives, and combination approaches improved bioavailability, tumor targeting, and efficacy. In vivo studies reported inhibition of tumor-growth and metastasis, improved pharmacokinetic profiles, and prolonged survival. However, no clinical trials evaluating AM in breast cancer patients have been reported to date. Overall, AM represents a promising preclinical candidate for breast cancer treatment. Nevertheless, standardized pharmacokinetic, toxicological, and efficacy studies, followed by well-designed clinical trials, are essential to facilitate its translation into clinical practice.

## 1. Introduction

Breast cancer remains one of the most prevalent malignancies and a major cause of cancer-related mortality among women worldwide. In 2022, approximately 2.3 million new cases and more than 666,000 deaths were reported globally, highlighting the continuing clinical burden of this disease [1,2,3]. Although major advances have been achieved in breast cancer management through surgery, chemotherapy, radiotherapy, endocrine therapy, targeted therapy, and immunotherapy, several therapeutic challenges remain unresolved. These include systemic toxicity, multidrug resistance, tumor recurrence, treatment-related adverse effects, and reduced quality of life among patients [4,5,6]. Therefore, the identification of safer and more effective therapeutic candidates, particularly those capable of modulating multiple cancer-related pathways, remains an important priority in breast cancer research.

Natural products have long been a valuable source of bioactive compounds for anticancer drug discovery due to their structural diversity and wide range of pharmacological activities. Among these compounds, α-mangostin (AM), a prenylated xanthone predominantly isolated from the pericarp of *Garcinia mangostana* L., has garnered significant scientific attention. Since its initial isolation, AM has been extensively studied for various biological activities, including antioxidant, anti-inflammatory, antimicrobial, cardioprotective, neuroprotective, and anticancer effects. In the realm of cancer research, AM has exhibited promising efficacy against a diverse array of cancer types, encompassing colorectal cancer, lung cancer, prostate cancer, leukemia, hepatocellular carcinoma, cervical cancer, osteosarcoma, and breast cancer [6,7,8]. In cancer models, AM has demonstrated its ability to regulate several molecular processes involved in carcinogenesis and tumor progression. These processes include apoptosis induction, cell cycle arrest, inhibition of PI3K/Akt signaling, suppression of angiogenesis and metastasis, modulation of oxidative stress, and mitochondrial dysfunction-mediated cell death [6,8,9,10,11]. These properties suggest that AM may act not only as a cytotoxic agent but also as a redox-modulating compound with potential relevance in antioxidant-based cancer therapy.

In breast cancer models, AM has shown promising activity against several molecular targets and signaling pathways. Previous studies have reported that AM can inhibit intracellular fatty acid synthase (FAS), induce apoptosis, regulate RXRα-AKT signaling, and interact with estrogen receptor alpha (ERα), thereby suppressing breast cancer cell proliferation and metastatic potential [8,9,12,13,14]. In addition, AM-mediated modulation of reactive oxygen species (ROS), mitochondrial signaling, and oxidative stress-related pathways indicates its potential role in controlling redox imbalance in breast cancer cells. Since oxidative stress contributes to breast cancer initiation, progression, therapeutic resistance, and metastatic behavior, compounds with dual antioxidant and anticancer mechanisms may provide meaningful translational value.

Despite its substantial pharmacological potential, the clinical translation of AM has been hindered by several inherent physicochemical and pharmacokinetic challenges, such as poor aqueous solubility, low bioavailability, rapid metabolism, and restricted systemic stability [6,15]. Consequently, recent studies have increasingly concentrated on not only assessing the direct anticancer activity of AM, but also on developing AM-based biomedical applications with the objective of enhancing its therapeutic efficacy. Various pharmaceutical and biomedical strategies, encompassing nano-formulations, polymeric nanoparticles, radiopharmaceuticals, lipid-based carriers, micellar systems, hydrogels, targeted delivery platforms, and combination-based approaches, have been investigated to augment stability, bioavailability, tumor selectivity, and therapeutic efficacy in breast cancer models [16,17,18,19,20,21,22,23,24,25,26].

Several reviews have discussed the anticancer potential of AM, including its role in breast cancer. However, most available reviews have primarily focused on its general pharmacological and molecular anticancer mechanisms, while limited attention has been given to the translational development of AM through integrated in silico, in vitro, and in vivo evidence. Therefore, this systematic review aims to provide a comprehensive evaluation of AM as a potential therapeutic candidate for breast cancer, with particular emphasis on antioxidant mechanisms, redox modulation, molecular targets, formulation strategies, and biomedical applications [27]. By synthesizing current preclinical evidence, this review highlights the opportunities and challenges associated with advancing AM toward future translational and clinical development in breast cancer therapy.

## 2. Methods

### 2.1. Study Design

**Figure 1:**
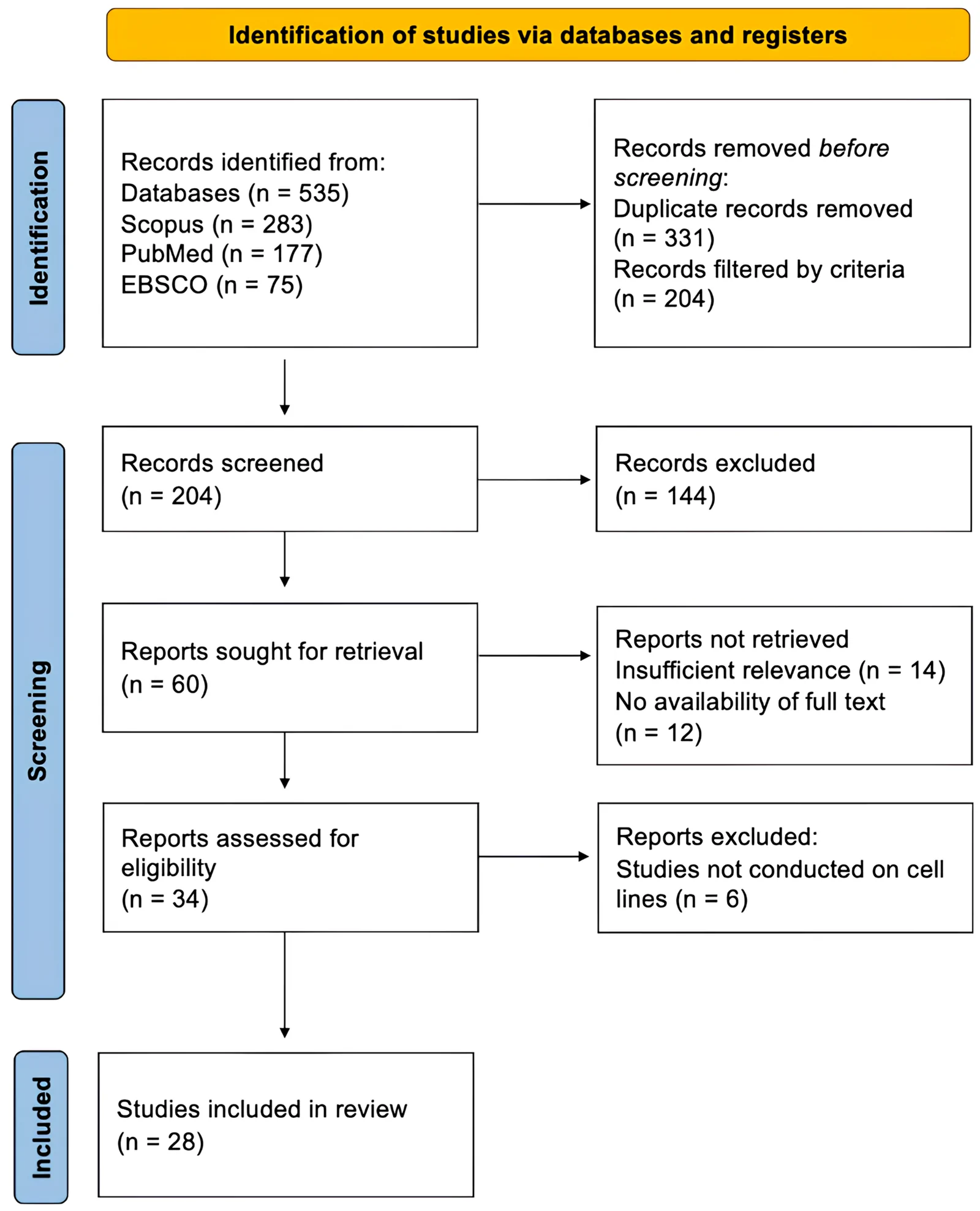
PRISMA flow diagram describing the study identification, screening, and inclusion process for studies evaluating alpha-mangostin in breast cancer models.

This systematic review was conducted to evaluate the preclinical evidence supporting α-mangostin (AM) as a potential therapeutic candidate for breast cancer. The review focused on studies investigating the anticancer activity, antioxidant and redox-modulating mechanisms, molecular targets, formulation strategies, and translational relevance of AM using in silico, in vitro, and in vivo approaches. The review process was guided by the Preferred Reporting Items for Systematic Reviews (PRISMA) 2020 guidelines [28]. The review protocol was not registered in a systematic review database. The study selection process is presented in Figure 1.

### 2.2. Eligibility Criteria

Original research articles were included if they investigated AM as a therapeutic candidate or preventive agent against breast cancer. Eligible studies were required to use in silico, in vitro, or in vivo experimental models and to report outcomes related to molecular mechanisms, antioxidant or redox regulation, formulation development, biomedical applications, pharmacological activity, or therapeutic efficacy of AM. Articles published in English between January 2019 and May 2026 were considered eligible. No restrictions were applied regarding breast cancer subtype, receptor status, cell line, animal model, or pharmaceutical formulation in order to provide a comprehensive overview of the preclinical development of AM in breast cancer research.

Studies were excluded if they were review articles, conference abstracts, editorials, commentaries, letters to the editor, book chapters, notes, or duplicate publications. Articles were also excluded when the title, abstract, or full text was not to the use of AM in breast cancer, if sufficient experimental data or extractable outcomes were unavailable, or if the experimental model fell outside the scope of this review.

### 2.3. Search Strategy

A systematic literature search was conducted in two electronic databases, Scopus and PubMed, to identify relevant studies published from January 2019 to May 2026. The search strategy was designed to retrieve studies related to AM and its biomedical application in breast cancer therapy. The following keyword combinations were used:

1. “alpha AND mangostin AND breast AND cancer”;
2. “alpha AND mangostin AND antioxidant”.

The search was limited to article titles, abstracts, and keywords, and the results were filtered according to the predefined inclusion and exclusion criteria.

All retrieved records were exported to EndNote Reference Manager for reference management and duplicate removal. The search strategy, database sources, search period, and selection process were documented to ensure transparency and reproducibility.

### 2.4. Study Selection

After duplicate removal, the remaining records were screened independently by two reviewers (BSM and JJ) based on titles and abstracts. Studies considered potentially relevant were subsequently assessed through full-text review according to the eligibility criteria. Any disagreement between reviewers during the selection process was resolved through discussion and consensus. Studies that fulfilled all inclusion criteria were included in the final qualitative synthesis. The complete identification, screening, eligibility assessment, and inclusion process are summarized in the PRISMA flow diagram.

### 2.5. Risk of Bias Assessment

The methodological quality and risk of bias of the included in vivo studies were assessed independently by three reviewers (BSM, JJ, and FP) using the Animal Research: Reporting of In Vivo Experiments (ARRIVE) guidelines [29]. The assessment encompassed critical reporting domains, including study design, sample size, inclusion and exclusion criteria, randomization, blinding, outcome measures, statistical methodologies, animal characteristics, experimental procedures, and reported results. Discrepancies in assessment were resolved through discussion until consensus was achieved.

Due to the substantial variation in experimental design, animal models, intervention types, outcome measures, and reporting quality among the included studies, a quantitative meta-analysis was not conducted. Instead, the findings were synthesized qualitatively.

### 2.6. Data Extraction

Data were extracted manually from eligible studies using a standardized data extraction form prepared in Microsoft Word. The extracted information included the type of AM modification or development strategy, modifying agents or formulation systems, experimental model, molecular or pharmacological targets, main findings from in silico, in vitro, and in vivo evaluations, and cited references.

The extracted data were categorized according to the biomedical or pharmaceutical development strategy, including radiolabeling, nanoformulation, structural modification, targeted delivery, and combination therapy. This categorization was used to compare findings across studies and to identify research trends, therapeutic mechanisms, translational opportunities, and remaining gaps in the development of AM for breast cancer therapy.

### 2.7. Data Synthesis

Due to methodological heterogeneity among the included studies, data synthesis was conducted narratively rather than statistically. Findings were categorized based on the experimental approach, specifically in silico, in vitro, and in vivo studies. Particular emphasis was placed on AM-related antioxidant mechanisms, redox modulation, molecular target interactions, cytotoxic and pro-apoptotic effects, formulation-related enhancements, pharmacokinetic behavior, tumor inhibition, and translational relevance. The synthesized findings were subsequently summarized in tables and discussed in relation to the current evidence supporting the development of AM as a potential therapeutic candidate for breast cancer.

## 3. Results

### 3.1. PRISMA Diagram and Data Extraction

A literature search identified 535 records from three electronic databases, comprising 283 records from Scopus, 177 records from PubMed, and 75 records from EBSCO.

After eliminating 331 duplicate records, 204 records remained for further evaluation. Of these, 144 records were excluded based on predefined filtering and screening criteria. The remaining 60 records were screened based on their titles and abstracts, resulting in the exclusion of 14 studies due to insufficient relevance to the scope of the review.

A total of 60 reports were sought for full-text retrieval. However, 26 full reports could not be retrieved. Consequently, 34 full-text articles were assessed for eligibility. Six studies were excluded because the experimental models were not based on human breast cancer cell lines. Ultimately, 28 studies met the inclusion criteria and were included in the qualitative synthesis. The study selection process is summarized in the PRISMA flow diagram shown in Figure 1.

### 3.2. General Characteristics of Included Studies

Twenty-nine studies investigated α-mangostin (AM) in breast cancer models that were related to the antioxidant mechanism. These studies employed various experimental approaches, including in silico, in vitro, and in vivo methods. The studies covered several biomedical and pharmaceutical development strategies, including radiolabeling, nanoformulation, structural derivatization, targeted delivery, and combination therapy.

AM is a xanthone compound isolated from the pericarp of the mangosteen fruit. Its IUPAC name, 1,3,6-trihydroxy-7-methoxy-2,8-bis(3-methylbut-2-en-1-yl)-9H-xanthen-9-one, indicates that AM is an 8-prenylated xanthone, distinguished by the presence of a C5-isoprenoid group attached to the xanthone core at the C-8 position. This compound appears as a yellowish powder and features side chains containing isoprene, methoxyl, and hydroxyl functional groups [30]. Based on its pKa values, α-mangostin (AM) exhibits weak acidic ionization behavior and poor aqueous solubility, which contribute to its limited oral bioavailability [16]. The physicochemical properties of AM are shown in Table 1.

The included studies evaluated AM using several breast cancer cell lines, including MCF-7, MDA-MB-231, SKBR3, T-47D, SUM-229PE, HCC1806, MBCDF, and 4T1 cells. These models represented different molecular characteristics of breast cancer, including hormone receptor-positive, HER2-positive, and triple-negative breast cancer models. Several studies also used animal models to evaluate tumor inhibition, biodistribution, pharmacokinetic behavior, metastasis suppression, and survival outcomes.

The extracted data were categorized according to the main development strategy, modifying agent, experimental model, molecular target, and major findings. A summary of the included studies is presented in Table 2.

### 3.3. Quality Assessment

**Figure 2:**
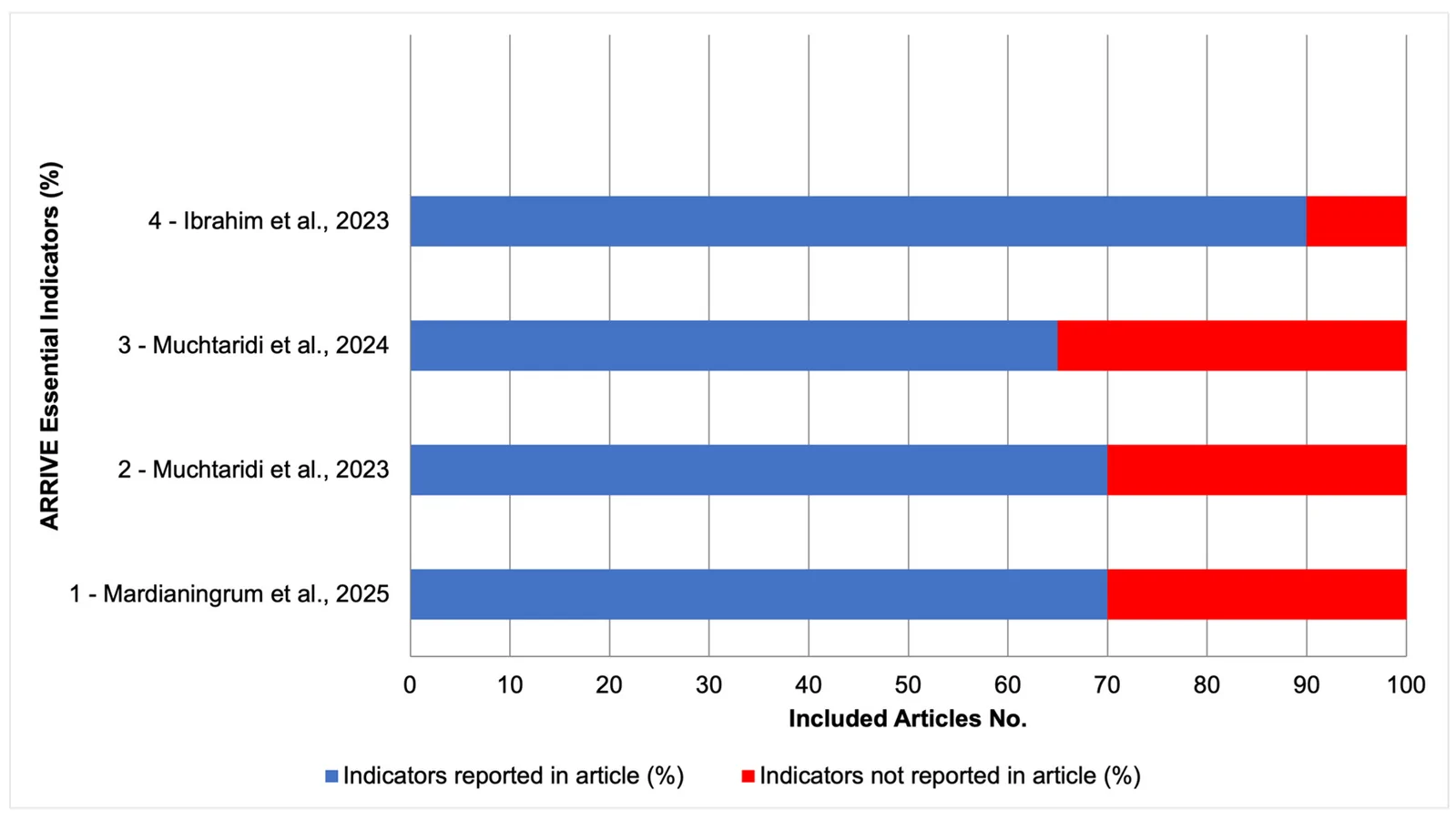
Quality assessment of in vivo data from AM in breast cancer [26,37,46,49].

The methodological quality of the included in vivo studies, assessed using the ARRIVE guidelines, is summarized in Figure 2. Overall, most studies adequately reported the study design, intervention procedures, outcome measures, and primary findings. However, several methodological limitations were identified, including incomplete reporting of randomization procedures, lack of blinding, limited justification of sample size, and insufficient information regarding inclusion and exclusion criteria [56]. These limitations should be considered when interpreting the strength of the preclinical evidence. The overall quality assessment is summarized in Figure 2.

### 3.4. Breast Cancer

Breast cancer is recognized as the most common type of cancer affecting women worldwide. According to the revised Paradigm II Model (2015), four major categories of causative factors have been identified: social (access to healthcare), environmental (exposure to chemicals), behavioral or lifestyle-related (alcohol consumption, smoking, obesity), and biological (genetic and hormonal factors). This model describes a multifactorial interaction, whereby various determinants simultaneously contribute to the initiation and development of breast cancer [57].

The pathogenesis of breast cancer is widely recognized as complex and has been associated with genetic mutations that disrupt cellular functions and trigger uncontrolled cell proliferation [58]. This process is influenced by several factors, such as mutations in tumor suppressor genes including *BRCA1*, *BRCA2*, *TP53*, and HER2. Among these, *BRCA1* and *BRCA2* mutations are genetic alteration most strongly associated with hereditary breast cancer susceptibility [59]. Inherited susceptibility is often attributed to germline mutations in moderate to high penetrance genes, including *CHEK2*, *TP53*, *BRCA1/2*, and *PALB2*. The inactivation of both alleles in these tumor suppressor genes initiates oncogenic signaling cascades [60]. Furthermore, hormonal factors, such as prolonged exposure to estrogen and reproductive-related events such as late menopause, nulliparity, abortion, and early menarche, have been identified as contributors to increased breast cancer risk [61].

In addition, unhealthy lifestyle factors have also been associated with increased breast cancer susceptibility. These include radiation exposure, physical inactivity, smoking, alcohol consumption, and disruption of circadian rhythm [62]. Circadian rhythm disruption has been reported to alter gene expression and interfere with the cell cycle, which may directly contribute to the initiation of malignancy. Such disruption suppresses melatonin secretion and accelerates inflammatory responses, thereby facilitating oncogenesis [63].

### 3.5. Current Treatment of Breast Cancer

Currently, breast cancer therapy has undergone rapid advancements. These developments have been driven by significant improvements in the accuracy of breast cancer detection and diagnosis. The emergence of novel therapeutic concepts and drugs has led to a paradigm shift toward personalized treatment approaches. In this context, diagnostic and therapeutic strategies are being optimized according to individual patient risk and specific molecular subtypes, thereby supporting the current era of oncology and resulting in more precise clinical outcomes in breast cancer management [59].

Current standard modalities for breast cancer include surgery, chemotherapy, radiotherapy, endocrine therapy, targeted therapy, and immunotherapy [64]. According to Sun et al. (2022), targeted therapies involving monoclonal antibodies (mAbs) against HER2, such as trastuzumab and pertuzumab, have been utilized as adjuvant treatments for HER2-positive and metastatic breast cancers [65]. Additionally, PARP inhibitors such as olaparib have been shown to exploit synthetic lethality in BRCA-mutated cancer cells by interfering with DNA repair mechanisms, thus selectively targeting malignant cells while sparing healthy ones [66]. Furthermore, bevacizumab (Avastin^®^), the first monoclonal antibody designed to inhibit angiogenesis, has demonstrated significant impact in cancer therapy [33].

Natural compounds, specifically vinca alkaloids such as vincristine, vinblastine, and vindesine, are widely employed in the treatment of breast cancer. Vinblastine stands out as one of the most extensively researched and commonly utilized vinca alkaloids. Typically administered at a dosage of 6 mg/m^2^, it exerts its therapeutic effect by binding to β-tubulin during metaphase, thereby inhibiting mitotic cell division [67]. Resveratrol has been extensively studied and is recognized for its ability to inhibit the expression of PGK1. PGK1 is an enzyme linked to accelerated cell proliferation and metastasis in cancer cells due to its involvement in the MYC and β-catenin signaling pathways. These findings suggest that natural compounds hold significant promise in the treatment of breast cancer [68] and support the continued exploration of natural-product-derived compounds, including α-mangostin, as potential therapeutic candidates.

### 3.6. Current Studies of Alpha Mangostin as Breast Cancer Treatment

**Figure 3:**
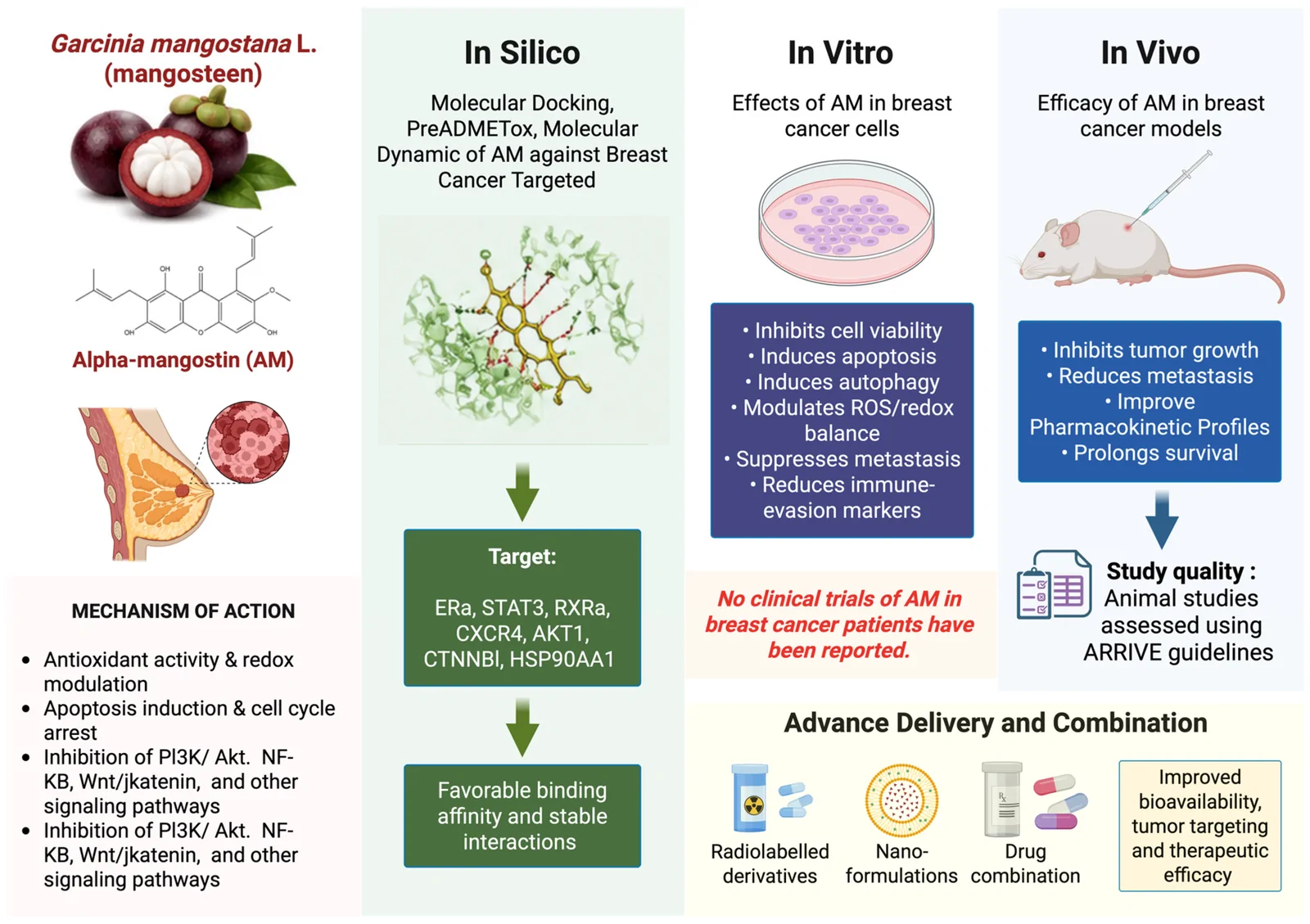
Overview of alpha-mangostin (AM) development as a potential therapeutic agent for breast cancer (created in BioRender. (2026) https://BioRender.com/vl9jcey. Accessed on 15 July 2026).

Figure 3 provides a concise overview of the current state of AM as a potential therapeutic candidate for breast cancer. Given the limitations and adverse effects associated with conventional breast cancer treatments, increasing attention has been directed towards natural bioactive compounds with multitarget anticancer properties. AM, a xanthone derivative isolated from *Garcinia mangostana* L., has demonstrated significant potential through various biomedical development strategies, including radiolabeling, nano-formulation, structural derivatization, and combination therapy approaches. These strategies have been investigated through integrated in silico, in vitro, and in vivo studies to enhance pharmacological activity, bioavailability, tumor selectivity, and therapeutic efficacy. The figure illustrates how computational prediction, experimental validation, and preclinical evaluation collectively contribute to the preclinical development of AM for breast cancer management.

#### 3.6.1. In Silico Studies of AM

In silico approaches refer to the use of computations in drug discovery and development studies [69]. This method aims to predict and characterize interactions between drug candidates and their molecular targets, thereby reducing the likelihood of failure during drug development [70]. These computational-based methods serve as an important starting point in drug discovery because they can reduce the cost, time, and attrition rates associated with conventional experimental approaches [71].

Several included studies used computational approaches to predict the interaction between AM and breast cancer-related molecular targets. Molecular docking and molecular dynamics simulations suggested that AM may interact with several proteins associated with breast cancer proliferation, survival, metastasis, and immune regulation. A study involving triple-negative breast cancer (TNBC) cells by Rivaldo and Chandra (2024) and Citra et al. (2023) employed molecular docking, pharmacophore modeling, and molecular dynamics simulation to demonstrate that AM interacts with receptors through multiple hydrogen and hydrophobic bonds [33,72]. The binding energies for all tested targets (AKT1, CTNNB1, HSP90AA1) were reported to be ≤−5.0 kcal/mol [33]. α-Mangostin exhibits significant potential as an anti-TNBC agent due to its ability to inhibit GSK3β. The molecular dynamics simulation, employing the molecular mechanics Poisson-Boltzmann and surface area (MM-PBSA) calculations method, reveals that α-mangostin exhibits a stronger affinity (with a ΔGTotal value of −114.463 kJ/mol) compared to the native ligand (with a ΔGTotal value of −75.158 kJ/mol) [72].

In another study, Zhu et al. 2021 reported that AM exhibited a binding energy of −10,235 kcal/mol toward the RXRα receptor (a protein that can cause uncontrolled tumor growth when hydrolyzed), and interacts with amino acid residues Arg-316, Ala327 and Leu433. The binding energy indicates that AM has a strong binding ability with the receptor [8]. These findings suggest a favorable interaction between AM and RXRα, although experimental validation is required to confirm its biological relevance.

Additional molecular docking studies demonstrated that AM exhibits a relatively high affinity for the CXCR4 receptor. CXCR4 is a protein implicated in cancer cell metastasis, and its expression levels are elevated in malignant cancers, including TNBC. A study conducted by Sarmoko et al. [35] revealed that AM possesses a lower binding energy (−12.0385 kcal/mol) compared to the native ligand (9.8801 kcal/mol). This finding suggests that AM has a greater potential to inhibit CXCR4 activity [35].

Nalla et al. (2023) evaluated the interaction between AM and Signal Transducer and Activator of Transcription 3 (STAT3), a protein implicated in cancer progression. Molecular docking analysis revealed that the binding energy of the AM-STAT3 complex was −3.362 kcal/mol, lower than that of the reference inhibitor, STX-0119 (−2.592 kcal/mol). This data suggests that AM possesses significantly enhanced potential activity compared to the standard. Molecular dynamic simulation further suggested that AM contributed to the stabilization of STAT3, with hydrogen bond formation involving the amino acid residues Tyr640, Met660, and Gly656 after a 100 ns simulation [14].

Research has also focused on AM derivatives. Muchtaridi et al. and Mardianingrum et al., identified that a derivative with the chemical structure C_28_H_31_NO_7_ exhibited stronger predicted interaction with ERα than the parent compound [73,74]. This derivative displayed a binding energy of −10.12 kcal/mol, whereas AM showed a binding energy of −9.05 kcal/mol. Similarly, three AM derivatives, AMB-1 (−9.84 kcal/mol), AMB-2 (−6.80 kcal/mol), and AMB-10 (−12.42 kcal/mol), exhibited lower binding free energies than AM (−1.77 kcal/mol) based on MM-PBSA calculation. Recognizing its promising potential through in silico studies, research on AM has been continued and expanded in various in vitro studies on several cell lines. The in silico results were validated by laboratory experiments, which demonstrated moderate activity (57–126 μM) for all derivatives in the in vitro assay [74].

#### 3.6.2. In Vitro Study of AM

In vitro studies involve experiments conducted under controlled conditions outside a living organism and represent an essential stage in drug discovery and development [75,76]. Findings obtained in vitro can provide predictive guidance for subsequent in vivo testing [77]. Various in vitro assays have been conducted, including real-time drug release [78], dissolution [79], bioavailability-related assessment [80], and cytotoxicity evaluation using cancer cell lines [81]. In the context of this review, in vitro studies were primarily used to evaluate the cytotoxic, pro-apoptotic, antioxidant, and anti-metastatic effects of AM in breast cancer cell models.

A study conducted by Cruz-Gregorio et al. (2023) evaluated the activity of AM on redox state, mitochondrial metabolism, and apoptosis of the 4T1 cell line. This cell line represents a model of stage IV breast cancer and can generate xenografted tumors in mice. AM reduces the viability of 4T1 cells, as determined by the MTT assay with an IC_50_ value of 13.98 μM. A decrease in catalase activity without significant changes in the activities of antioxidant enzymes such as superoxide dismutase (SOD), glutathione peroxidase (GPx), glutathione S-transferase (GST), and glutathione reductase (GR) was observed following treatment with 18 μM AM. In addition, AM reduced basal respiration, oxidative phosphorylation, and respiratory control in 4T1 cells, suggesting the induction of mitochondrial dysfunction. The study also demonstrates that AM induced apoptosis and promoted cell death activity in 4T1 cells [36].

In a separate study, Meylina et al. (2023) investigated the potential of formulating AM with hyaluronic-acid-coated chitosan-based nanoparticles for targeted treatment against the MCF-7 cell line. Cytotoxicity tests revealed that AM exhibited an IC_50_ of 5.27 μg/mL, while the hyaluronic-acid-coated chitosan formulation demonstrated an IC_50_ of 4.37 μg/mL, suggesting enhanced cytotoxic activity following nanoformulation [50]. Similarly, Muchtaridi et al. (2023) reported that AM encapsulated within chitosan/alginate polymer-based nanoparticles exhibited an IC_50_ value of 2.744 μg/mL compared with 8.350 μg/mL for pure AM. These findings suggest that nanoparticle-based formulations may enhance the antiproliferation activity of AM against breast cancer cells. Further in vivo testing can be conducted to elucidate the impact of compounds on animal models [49].

#### 3.6.3. In Vivo Studies of AM

In vivo drugs are recognized as a critical step in the discovery and development of new therapeutic agents, particularly in oncology. Through in vivo testing, pharmacodynamic and pharmacokinetic parameters, systemic toxicity, and therapeutic efficacy can be comprehensively evaluated within a whole-organism context, which cannot be fully achieved through in vitro studies alone [82]. The in vivo method is considered essential in drug research and development, involving the direct administration of test compounds into animal models such as mice, rats, rabbits, and others, with dosages adjusted according to experimental protocols. At predetermined endpoints, animals are euthanized humanely and biological samples such as blood or tissues are collected for further analysis [83].

In vivo evaluations have been conducted using experimental animals, primarily rats and mice. A study by Shibata et al. [84] utilized BJMC3879luc2-induced mammary tumor-bearing rats and demonstrated that treatment with 20 mg/kg AM effectively reduced tumor size and suppressed metastatic progression by delaying the spread of tumor cells to other lymphatic nodes. Moreover, the treatment significantly prolonged the survival of the rats without any noticeable changes in body weight throughout the 6-week treatment period. Furthermore, a recent study reported the antineoplastic activity of AM at doses of 30 mg/kg and 60 mg/kg in LA7-induced breast tumor-bearing mice. The treatment significantly suppressed mitotic activity commonly observed in breast adenocarcinoma and reduced the levels of tumor biomarkers such as CEA and CA15-3. Histopathological evaluation further revealed that AM induced apoptosis, which was associated with upregulation of p53 and downregulation of PCNA expression in tumor tissues [37]. These findings collectively highlight the antitumorigenic potential of AM in breast cancer, suggesting that its mechanism of action involves the modulation of key molecular pathways regulating cell proliferation and apoptosis.

In addition, several in vivo studies have further documented the antitumor properties and potential applications of AM in animal models of cancer. According to Mardianingrum et al. (2025), radioactive labeling of [^131^I]I-AM-Co was performed through electrophilic aromatic substitution using bis(α-mangostin)-cobalt (II) (AM-Co) as the precursor compound in an animal model. Pharmacokinetic profiling in Sprague-Dawley rats and biodistribution studies in BALB/c mice demonstrated that the compound was rapidly distributed and cleared, as indicated by its short half-life (t½) of 0.33 ± 0.17 h, suggesting a rapid redistribution from blood to tissues. Furthermore, the area under the curve (AUC) was found to be five times greater than that of iodine-131, suggesting improved systemic exposure of [^131^I]I-AM-Co. However, iodine-131 alone exhibited longer retention appears inconsistent with the higher AUC reported for [^131^I]I-AM-Co and should be clarified [46]. In a separate study, Muchtaridi et al. (2024) evaluated radiolabeled [^125^I]I-AM in estrogen receptor alpha(Erα)-positive breast cancer models. The compound was administered via intravenous injection of 100 µL of 1% DMSO in PBS (at a dose of 7.4 kBq/mouse, adjusted by body weight) into 5-week-old male ddY mice. The results indicated significant tumor accumulation, although partial deiodination of the compound was still observed [26].

Furthermore, Muchtaridi et al. (2023) evaluated a nanoparticle polymer formulation consisting of AM combined with chitosan and alginate (referred to as NANO-AMCAL). It was found that NANO-AMCAL containing 0.33 mg of AM exhibited a therapeutic effect approximately three-fold greater than that of 20 mg of pure AM. Additionally, NANO-AMCAL administered at a dose of 20 mg was shown to maintain body weight in tumor-bearing animals and reduce tumor volume by 17.43% [49]. Collectively, these findings indicate that in vivo administration of AM, whether in its free form or modified as a nanoparticle or radiolabeled compound, demonstrates promising antitumor activity by inhibiting tumor progression and reducing tumor size in breast cancer models.

Oral administration of α-mangostin significantly suppressed primary tumor growth and reduced lymph node metastasis in an immunocompetent mouse model of metastatic mammary carcinoma. These antitumor effects were associated with increased apoptosis, inhibition of angiogenesis, and reduced lymphatic invasion. Furthermore, α-mangostin induced apoptosis in breast cancer cells through caspase activation, disruption of the mitochondrial membrane potential, increased DNA fragmentation (TUNEL-positive cells), and G1-phase cell cycle arrest [85].

### 3.7. Antioxidant and Redox-Modulating Mechanisms of Alpha-Mangostin in Breast Cancer

**Figure 4:**
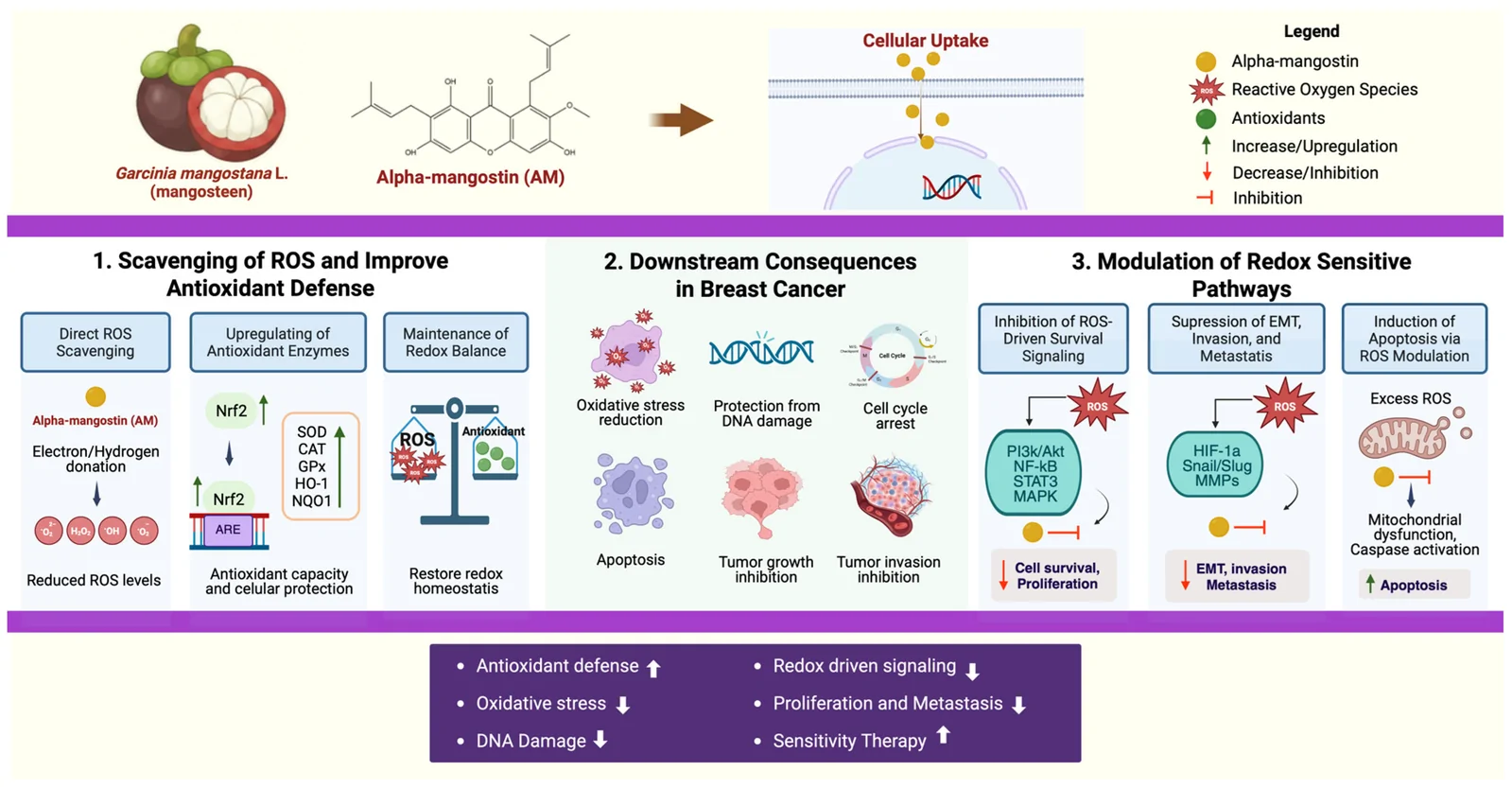
Antioxidant and redox-modulating mechanisms of α-mangostin in breast cancer cells (created in BioRender. (2026) https://BioRender.com/vl9jcey. Accessed on 15 July 2026).

As shown in Figure 4, AM may exhibit anticancer effects in breast cancer through a multifaceted redox-modulating mechanism. Initially, it enhances antioxidant defense by directly scavenging ROS and activating the nuclear factor Nrf2/ARE pathway. This activation leads to the upregulation of key antioxidant enzymes such as SOD, CAT, GPx, and HO-1, thereby restoring redox homeostasis and mitigating oxidative damage. Concurrently, it has been reported to modulate redox-sensitive oncogenic signaling by inhibiting pathways including the PI3K/Akt, NF-κB, STAT3, and MAPK pathways. Additionally, it suppresses EMT-associated factors such as HIF-1α, Snail/Slug, and MMPs, resulting in reduced proliferation, survival, invasion, and metastasis. In tumor cells with elevated basal ROS, AM further induces excessive oxidative stress, disrupting mitochondrial function to activate caspase-dependent apoptotic pathways and also inhibiting cancer cells’ migration [35]. Collectively, these mechanisms suggest that α-mangostin may contribute to the suppression of tumor growth and enhancement of therapeutic sensitivity in preclinical breast cancer models.

#### 3.7.1. Oxidative Stress and Redox Signaling in Breast Cancer

Oxidative stress is a pivotal factor in the initiation and progression of breast cancer [86,87]. ROS, such as superoxide anion, hydrogen peroxide, and hydroxyl radicals, are continuously generated during cellular metabolism [88,89]. Under physiological conditions, ROS play an important role in intracellular signaling, proliferation, and immune responses [90,91]. However, excessive ROS production or compromised antioxidant defense mechanisms result in oxidative stress, leading to DNA damage, genomic instability, aberrant signaling pathways, and tumor development [92,93].

Breast cancer cells frequently exhibit elevated ROS levels due to increased metabolic activity, mitochondrial dysfunction, oncogene activation, and interactions with the tumor microenvironment [94,95]. Persistent oxidative stress activates multiple oncogenic pathways, including PI3K/Akt, NF-κB, STAT3, HIF-1α, and MAPK signaling, thereby promoting proliferation, angiogenesis, invasion, metastasis, and therapeutic resistance [96,97]. Consequently, modulation of redox homeostasis has emerged as an attractive therapeutic strategy in breast cancer management [98,99,100].

El Gaafary and co-workers reported that α-mangostin, γ-mangostin, 9-hydroxycalabaxanthone (9-HCX), and garcinone E from mangosteen pericarp exhibited cytotoxic activity against several human cancer cell lines, including triple-negative breast cancer MDA-MB-231 cells. These compounds target cancer cell mitochondria by inhibiting respiratory chain complexes II and III, reducing OXPHOS and ATP production while increasing proton leak and mitochondrial superoxide generation. This resulted in mitochondrial membrane permeabilization and caspase 3/7-mediated apoptosis. These findings suggest that AM and related xanthones may exhibit anticancer effects, at least in part, through disruption of mitochondrial respiration and redox regulation [39].

#### 3.7.2. Antioxidant Properties of Alpha-Mangostin

AM has been widely recognized as a natural antioxidant capable of modulating oxidative stress through multiple molecular mechanisms [101]. Previous studies have demonstrated that AM enhances the endogenous antioxidant defense system by increasing the activity or expression of antioxidant enzymes, including superoxide dismutase (SOD), catalase (CAT), and glutathione peroxidase (GPx), while also restoring intracellular glutathione (GSH) levels and activating the nuclear factor erythroid 2-related factor 2/heme oxygenase-1 (Nrf2/HO-1) signaling pathway [102,103,104,105]. In non-cancer disease models, AM consistently attenuated oxidative stress by reducing intracellular reactive oxygen species (ROS), lipid peroxidation, and inflammatory mediators, while preserving mitochondrial function and cellular redox homeostasis [102,103,104,106].

The antioxidant activity of AM is primarily attributed to its polyphenolic xanthone structure, which enables free radical scavenging and modulation of redox-sensitive signaling pathways [101]. Increasing evidence indicates that AM exerts its antioxidant effects, at least in part, through activation of the nuclear factor erythroid 2-related factor 2 (Nrf2)/Kelch-like ECH-associated protein 1 (Keap1) signaling pathway, a master regulator of cellular antioxidant defense [104,105,107]. Upon activation, Nrf2 translocates into the nucleus and binds to antioxidant response elements (AREs), thereby promoting the transcription of cytoprotective genes, including heme oxygenase-1 (HO-1), NAD(P)H quinone oxidoreductase-1 (NQO1), and other antioxidant enzymes that protect cells against oxidative injury [104,105,107,108].

Ibrahim et al. demonstrated that α-mangostin attenuated oxidative stress in LA7-induced mammary tumors by enhancing endogenous antioxidant defenses, including SOD, CAT, and GPx activities, while reducing malondialdehyde (MDA) levels, suggesting that its antioxidant properties may contribute to its antitumor effects in breast cancer [37]. Similar antioxidant mechanisms have also been reported in non-cancer disease models. Fang et al. demonstrated that α-mangostin protected against light-induced retinal injury by reducing photoreceptor degeneration, retinal pigment epithelial (RPE) cell apoptosis, intracellular ROS, and MDA levels, while restoring SOD, GPx, and GSH. These effects were mediated by Nrf2 nuclear translocation, HO-1 upregulation, PKC-δ activation, and suppression of MAPK signaling, including ERK1/2, JNK, and p38 phosphorylation, thereby preserving cellular redox homeostasis [102].

#### 3.7.3. Pro-Oxidant Activity of Alpha-Mangostin in Breast Cancer Cells

Interestingly, AM exhibits a paradoxical dual role in redox regulation [6,36,101]. While acting as an antioxidant in normal tissues, AM has been reported to function as a selective pro-oxidant in breast cancer cells [36,39,109]. Several studies included in this review reported increased intracellular ROS accumulation following AM treatment in breast cancer cell lines [15,35,36]. Elevated ROS levels subsequently were associated with mitochondrial dysfunction, loss of mitochondrial membrane potential, cytochrome c release, caspase activation, and apoptotic cell death [12,13,36,110,111,112].

This selective induction of oxidative stress appears to be one of the principal mechanisms underlying AM-mediated anticancer activity. AM has also been reported to suppress STAT3 signaling [14,113], inhibit CXCR4-mediated migration [35], reduce HIF-1α expression [114], modulate epithelial–mesenchymal transition (EMT) [115,116], and enhance sensitivity to chemotherapeutic agents [22,40,41,42,117]. Together, these findings indicate that AM exerts anticancer effects through the coordinated regulation of oxidative stress, redox-sensitive signaling pathways, and apoptosis-related mechanisms.

#### 3.7.4. Translational Significance of Redox Modulation by Alpha-Mangostin

The dual antioxidant and pro-oxidant properties of AM present a unique therapeutic opportunity [6,101]. Unlike conventional antioxidants that may inadvertently protect cancer cells, AM has been reported to maintain redox balance in normal tissues while selectively inducing oxidative stress in malignant cells [36,39,101,102]. This differential activity may contribute to improved therapeutic selectivity and reduced toxicity. Ruankham et al. demonstrated that AM protected SH-SY5Y cells against H_2_O_2_-induced oxidative stress by reducing cell death and modulating apoptosis and antioxidant-related proteins, including BAX, caspase-3/7, BCL-2, CAT, and SOD2. The study also suggested the involvement of the SIRT1/3-FOXO3a pathway in the cytoprotective effects of AM [106]. Although these findings were obtained in a neuroprotection model, they provide additional mechanistic support for the redox-modulating properties of AM.

Recent advancements in nanoparticle-based delivery systems, targeted formulations, and radiolabeled AM derivatives further support its potential translational relevance. Enhanced bioavailability, tumor accumulation, and pharmacological efficacy observed in preclinical studies suggest that AM warrants further investigation as a candidate for redox-based therapeutic strategies in breast cancer.

## 4. Discussion

Since its initial isolation and identification from the mangosteen plant in 1855, the therapeutic potential of alpha-mangostin (AM) has been extensively explored. Advances in medicine have highlighted AM as a bioactive compound with potential applications in various human diseases. Over the past decade, AM has been rigorously investigated across multiple therapeutic targets. Extensive research has also focused on structural modification and formulation to optimize its effectiveness and reduce the potential toxicity or side effects associated with AM.

Based on previous acute toxicity studies, AM exhibits lower toxicity following oral administration than intraperitoneal administration. Toxicity is also influenced by several factors, including dose, route of administration, and formulation [56]. In an in vivo zebrafish study, AM caused mortality and developmental abnormalities during embryonic development at 72 h after administration, with an LC_50_ of 5.75 ± 0.26 μM [118]. These findings are supported by another study showing that pure AM was more toxic to embryos than when it was present in crude xanthone extract [119].

Molecular docking and molecular dynamics simulation studies have shown that AM, as well as its derivatives, has affinity for several molecular targets, including AKT1, CTNNB1, HSP90AA1, hERα, RXRα, STAT3, and CXCR4 [8,14,26,33,43,44,47,72,73,74,120]. These data are supported by negative binding energies and hydrogen bond formation involving the phenolic hydroxyl groups of AM. Hydrogen bonds have been reported to involve the phenolic hydroxyl groups located at the C-1, C-3, or C-6 positions of AM. Weaker interactions, including π–π, π-sigma, π-alkyl, and van der Waals interactions, were also observed with multiple amino acid residues, indicating stable ligand–receptor binding. Interestingly, several AM derivatives exhibited better docking scores than the parent compound, although they interacted with a greater number of amino acid residues during molecular docking analyses [47,73,74,120]. These findings suggest that AM and its derivatives possess favorable binding affinity toward multiple therapeutic targets relevant to breast cancer.

At the cellular level, most studies have reported that AM suppresses proliferation and induces apoptosis, although the dominant pathways described vary across models [8,12,13,14,31,34,36,40,42,44]. In MDA-MB-231 cells, the effects of AM have been associated with modulation of PI3K/Akt, cyclin D1, mitochondrial dysfunction, and oxidative stress [8,36,44], whereas in MCF-7 cells, its pro-apoptotic effects have also been shown to involve upregulation of MOAP-1, interaction with BAX, downregulation of BCL-XL, cytochrome *c* release, and caspase activation [12,13,34,110,121]. In addition, AM has been reported to suppress STAT3 activation and reduce hnRNP-A1 expression, which has subsequently been linked to disrupted PKM2 regulation as well as reduced EMT potential, migration, and invasion [14,122], while other studies have shown suppression of CXCR4 transcription through increased ROS [35].

Changes in ROS level indicate the ability of alpha-mangostin (AM) to modulate oxidative processes within the cell, suggesting that AM functions as a redox-active compound that can act as either an antioxidant or a pro-oxidant depending on cell condition. ROS play a role in cellular signaling, however, when present in excessive amounts, they contribute to oxidative stress, biomolecular damage, mitochondrial dysfunction, and ultimately cell death. In breast cancer models, particularly 4T1 and MDA-MB-231, AM more frequently exhibits pro-oxidant effects, as indicated by induced oxidative damage, increased ROS levels, decreased catalase activity, oxidative stress associated with mitochondrial uncoupling, elevated H_2_O_2_, and increased mitochondrial superoxide. In addition to these ROS-related effects, AM has also been associated with reduced basal respiration, decreased efficiency of respiratory chain complexes, reduced OXPHOS and ATP production, and increased mitochondrial membrane permeabilization or depolarization, which lead to apoptosis. In contrast, in mammary tumor in vivo models, as well as several non-cancer models, AM has more often been associated with increased SOD, GPx, CAT, and GSH levels, along with decreased LPx, MDA, ROS, or H_2_O_2_, reflecting antioxidant activity and cytoprotective effects against oxidative stress.

Combination-based development has also shown the potential to increase sensitivity in breast cancer cell lines [32,34,35,41,49]. Combination with doxorubicin reduced cell viability and inhibited RALDH activity associated with stemness and resistance; combination with 4-hydroxytamoxifen reduced *CCND1* expression; and combination with 5-FU increased the proportion of cells in the subG1 phase. Meanwhile, a combination with palmitic acid induced autophagy through FAS inhibition and endoplasmic reticulum stress [49,123], whereas a combination with nab-paclitaxel increased caspase 3/7 activity and cytotoxicity. Nevertheless, these findings remain very limited to preclinical models and therefore still require further investigation regarding their effectiveness.

The development of nano-formulations has improved the performance of AM compared with its free or crystalline forms, particularly through increased solubility, cellular uptake, controlled release, and cytotoxicity [15,38,39,48,84]. NANO-AMCAL was reported to have higher cytotoxicity in MCF-7 cells and also reduced tumor volume and Bcl-2 expression in in vivo models, while chitosan/HA-, chitosan–folic acid-, and chitosan/κ-carrageenan-based formulations consistently lowered IC_50_ compared with free AM [39,48,84]. However, differences in efficacy among formulations remain, which appear to be influenced by particle size, chitosan molecular weight, the presence of targeting ligands such as folic acid, as well as HA coating that facilitates CD44-mediated endocytosis and pH-responsive release [48,53]. Thus, delivery systems represent one of the further development strategies that may be utilized to optimize the effectiveness of AM.

Radioiodination studies of AM have also shown promising results for theranostic applications and improved the stability of AM-based compounds [26,46]. Both [^125^I]I-AM and [^131^I]I-AM-Co demonstrated high cellular uptake and tumor accumulation [26,46]. However, partial deiodination was observed for [^125^I]I-AM, which may reduce intracellular isotope retention [26]. In contrast, [^131^I]I-AM-Co exhibited higher plasma exposure and slower diffusion from the bloodstream to organs than free iodine, indicating improved in vivo stability [46]. These findings suggest that structural modification of AM may overcome some of its pharmacokinetic limitations, although direct comparison between studies remains difficult because of differences in radioisotopes, animal models, and biodistribution protocols [26,46].

## 5. Author Perspective

Based on the comprehensive analysis conducted by the authors across in silico, in vitro, and in vivo approaches, alpha-mangostin (AM) has emerged as a promising preclinical candidate for breast cancer treatment. These effects have been associated with interactions involving several molecular targets implicated in cancer cell proliferation and metastasis, including human estrogen receptor (hER), retinoid X receptor (RXR), and signal transducer and activator of transcription 3 (STAT3). A general concordance between in silico prediction and in vitro findings was observed, with molecular docking studies reporting lower binding energies relative to reference ligands, suggesting favorable ligand–target interactions that warrant further experimental validation.

Consequently, the authors conclude that the findings obtained from studies evaluating AM, both in its natural form and as modified formulations or derivatives, across various preclinical stages highlight its potential as a candidate for further translation development. However, the current evidence remains limited to in vitro and in vivo, so its therapeutic relevance in humans cannot yet be established. Further studies are needed to clarify its safety, optimal formulation, bioavailability (how well a compound is absorbed and reaches tissues), and clinical effectiveness before translation into human application. In addition, this systematic review was limited by the predefined eligibility criteria and search strategy; therefore, other developments related to the use of AM as a potential anti-breast cancer agent may not have been captured. Further validation through pharmacokinetic, toxicological, and clinical investigations is required to establish the safety and efficacy of AM in human subjects.

## 6. Conclusions and Future Perspective

Current evidence from in silico, in vitro, and in vivo studies suggest that alpha-mangostin (AM) possesses considerable potential as a natural lead compound for breast cancer therapy. Beyond its direct anticancer activity, AM exhibits unique antioxidant and redox-modulating properties that may contribute to the regulation of oxidative stress, suppression of oncogenic signaling pathways, induction of apoptosis, and inhibition of tumor progression. The ability of AM to function as both an antioxidant and a selective pro-oxidant in cancer cells represents a particularly attractive characteristic for redox-based therapeutic strategies.

Advances in nanoparticle delivery systems, targeted formulations, combination therapies, and radiolabeled derivatives have further highlighted the potential translational relevance of AM by improving its bioavailability, tumor selectivity, and therapeutic efficacy in preclinical models. Nevertheless, significant challenges remain, including limited pharmacokinetic data, poor aqueous solubility, and the absence of clinical trials. Future studies should focus on elucidating the precise redox-regulatory mechanisms of AM, optimizing formulation strategies, evaluating long-term safety profiles, and conducting well-designed clinical investigations. Collectively, the available evidence supports AM as a promising candidate for future precision oncology approaches targeting oxidative stress and redox signaling in breast cancer.

## Figures and Tables

**Table 1:** Physicochemical properties of AM.

Chemical structure & formula	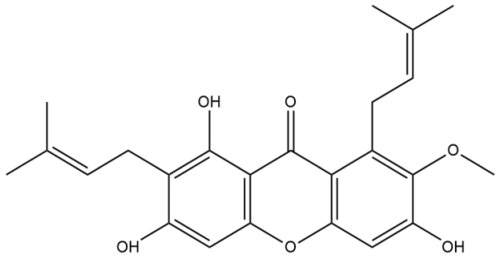 C_24_H_26_O_6_
Molecular weight	410.46 g/mol [6]
Melting point	180–181 °C [6]
Solubility	Poorly soluble in water; soluble in ethanol, methanol, and dimethyl sulfoxide (DMSO) [6]

**Table 2:** Data extraction of studies investigating alpha-mangostin in breast cancer models.

No.	Type of Modification	Modifying Agents	Result			**Reference**
			In Silico	In Vitro	In Vivo	
1	pure AM	-	-	Induced endoplasmic reticulum stress and autophagy in MDA-MB-231 cells.	-	[31]
2	pure AM	-	-	Induced oxidative damage and oxidative stress associated with mitochondrial uncoupling and higher H_2_O_2_ production in 4T1 cells.	-	[32]
3	pure AM	-	AKT1, CTNNB1, and HSP90AA1 were determined to be the most integral target proteins.	-	-	[33]
4	pure AM	-	Higher binding energy to STAT3 compared to the standard STX-0119. H-Bonds interaction with Tyr640, Met660 and Gly656.	Cytotoxic activity by a 24 h treatment in MCF-7 and MDA-MB-231 cell lines.	-	[14]
5	pure AM		Molecular docking study show that AM has a strong binding energy with RXRα and interaction with amino acid residues such as ARG-316, ALA327, and LEU-433.	× Invasion and metastasis in MDA-MB-231 cell lines by down-regulate the expression of cyclin D1.	-	[8]
6	pure AM		-	× MCF-7 cell lines proliferation. ↑ MOAP-1 tumor receptor activity. ↑ Activated-BAX. ↓ Expression of BCL-XL.	-	[34]
7	pure AM	-	↓ Gibbs energy towards the CXCR4	× MDA-MB-231 cell lines proliferation Modulation of ROS levels.	-	[35]
8	pure AM	-	Induced apoptosis in 4T1 cells by lowering Bcl-2 expression and caspase 3 cleavage. Induced oxidative damage by suppressing protective antioxidant enzymes and triggering a surge in harmful ROS.			[36]
9	pure AM	-	Induced apoptosis and inhibits metastasis of breast cancer cells by regulating the downstream effectors of the PI3K/AKT signaling pathway through the degradation of RXRα/tRXRα.			[8]
10	pure AM	-	-	-	↑ SOD, GPx, CAT and lower LPx levels compared to mammary tumor mice control	[37]
11	Combination	AM & Doxorubicin	-	↓ IC_50_ compared to single-agent treatment in MCF-7 cell lines by inhibiting RALDH activity.	-	[38]
12	Combination	Other xanthone (γ-mangostin and garcinone E)	-	× MDA-MB-231 cell lines proliferation. × Respiratory chain complexes II and III. ↓ OXPHOS and ATP production. ↑ Proton leak. ↑ Mitochondrial superoxide, promotes membrane permeabilization, and triggers caspase 3/7-mediated apoptosis.	-	[39]
13	Combination	AM & 4-hydroxytamoxifen	-	↑ Antiproliferation ability in MCF-7 and T-47D cell lines than each compound alone without combination.	-	[40]
14	Combination	AM & nab-paclitaxel	-	× MDA-MB-231 and MCF-7 cell lines proliferation. ↑ Caspase 3/7 activity in both cell lines.	-	[41]
15	Combination	AM & 5-Fluorouracil	-	↑ Cell proliferation activity and acted synergistically in SUM-229PE, HCC1806, MBCDF-D5, MBCDF, and T-47D cell lines than each compound alone.	-	[42]
16	Combination	AM & chitosan were conjugated with trastuzumab	↑ Binding score against HER2 and the formation of hydrogen bonds compared to AM alone	-	-	[43]
17	Combination	Lithium chloride	AM binds favorably to β-catenin and LRP6, with a higher affinity.	↓ Wnt transcriptional targets CCND1 and MYC in MDA-MB-231 cells.	-	[44]
18	Combination	Nanomedicine with GSH-responsive loaded shikonin	-	-	↓ Expressions of CAF markers α-SMA and FAP-α	[45]
19	Radiopharmaceutical	The synthesis of bis(α-mangostin)-cobalt (II) (AM-Co) was carried out via electrophilic aromatic substitution.	-	↑ Intake of Iodine-131 alone during the incubation period.	↑ Distribution and clearance while Iodine-131 has higher retention	[46,47]
20	Radiopharmaceutical	Iodine-125 radiolabeled compound ([^125^I]I-AM)	↑ Binding affinity toward ERα	↑ Cellular uptake of Iodine-125 in MCF-7 cell lines with time up to 1 h and then it decreases. Binding Site AM dengan Era serupa dengan tamoxifen namun berbeda dengan estradiol.	Significant tumor accumulation observed despite partial deiodination of the compound.	[26]
21	Radiopharmaceutical	Iodine-131 radiolabeled compound ([^131^I]I-AM)	-	AM found to be viable to MCF-7 cell line.	*-*	[48]
22	Polymeric nanoparticles	AM—Chitosan Alginat (NANO-AMCAL)	-	Solubility and effectiveness against MCF-7 cell lines.	↑ therapeutic effect and was comparable to tamoxifen. 17.43% reduction in tumor volume by day 14.	[49]
23	Nanoparticles	Chitosan—Hyaluronic acid	-	↑ Cytotoxic effect against MCF-7.	-	[50]
24	Nanoparticles	Folate-conjugated chitosan	-	↓ IC_50_ against MCF-7 cell lines increases the cytotoxicity against MCF-7 cell lines.	-	[51]
25	Nanoparticles	Low molecular weight chitosan nanoparticles (LMW NPs) and high molecular weight chitosan nanoparticles (HMW NPs)	-	↓ IC_50_ against MCF-7 cell lines in both LMW NPs and HMW NPs.	*-*	[52]
26	Nanoparticles	CD44-ThioaptAMer-tagged Nanoparticles	-	↓ HIF-1α modulation with lower concentration in MCF-7 cell line.	-	[53]
27	Nanoparticles	Chitosan-Kappa carrageenan (AM-Ch/Cr)	-	↓ IC_50_ in MCF-7 cell lines.	-	[54]
28	Liposomal nanocarrier dequalinum	A mitochondria-targeted nanoplatform using dequalinium-modified liposomal nanocarrier containing α-M for suppressing the growth of TNBC	-	↑ Cellular uptake. ↑ Mitochondrial accumulation. ↓ Viability of TNBC cells.	-	[55]

Note: ↑ increase; ↓ decrease; × inhibition; BAX (Bcl-2-associated X protein); Bcl-XL (B-cell lymphoma-extra-large/Bcl-2-like protein 1); ROS (reactive oxygen species); MOAP-1 (modulator of apoptosis 1); SOD (superoxide dismutase); GPx (glutathione peroxidase); CAT (catalase); LPx (lipid peroxide/lipid peroxidation); RALDH (retinaldehyde dehydrogenase); OXPHOS (oxidative phosphorylation); ATP (adenosine triphosphate); CAF (cancer-associated fibroblasts).

## Data Availability

No new data were created or analyzed in this study. Data sharing is not applicable to this article.
